# Insulin Resistance in HIV-Patients: Causes and Consequences

**DOI:** 10.3389/fendo.2018.00514

**Published:** 2018-09-05

**Authors:** Marcelo N. Pedro, Guilherme Z. Rocha, Dioze Guadagnini, Andrey Santos, Daniela O. Magro, Heloisa B. Assalin, Alexandre G. Oliveira, Rogerio de Jesus Pedro, Mario J. A. Saad

**Affiliations:** ^1^Department of Internal Medicine, Faculty of Medical Sciences, State University of Campinas-UNICAMP, Campinas, Brazil; ^2^Department of Surgery, Faculty of Medical Sciences, State University of Campinas-UNICAMP, Campinas, Brazil; ^3^Biosciences Institute, São Paulo State University (UNESP), Rio Claro, Brazil

**Keywords:** insulin resistance, diabetes, HIV, CVD, cART, LPS

## Abstract

Here we review how immune activation and insulin resistance contribute to the metabolic alterations observed in HIV-infected patients, and how these alterations increase the risk of developing CVD. The introduction and evolution of antiretroviral drugs over the past 25 years has completely changed the clinical prognosis of HIV-infected patients. The deaths of these individuals are now related to atherosclerotic CVDs, rather than from the viral infection itself. However, HIV infection, cART, and intestinal microbiota are associated with immune activation and insulin resistance, which can lead to the development of a variety of diseases and disorders, especially with regards to CVDs. The increase in LPS and proinflammatory cytokines circulating levels and intracellular mechanisms activate serine kinases, resulting in insulin receptor substrate-1 (IRS-1) serine phosphorylation and consequently a down regulation in insulin signaling. While lifestyle modifications and pharmaceutical interventions can be employed to treat these altered metabolic functions, the mechanisms involved in the development of these chronic complications remain largely unresolved. The elucidation and understanding of these mechanisms will give rise to new classes of drugs that will further improve the quality of life of HIV-infected patients, over the age of 50.

## Introduction

The introduction and evolution of antiretroviral drugs over the past 25 years has completely changed the clinical prognosis of HIV-infected patients (1). These drugs have transformed the disease into a chronic condition, and increased life expectancy, which is similar to the general uninfected population (2). Nowadays, the deaths of HIV-infected individuals, who appropriately follow their therapy regimen, are related to non-communicable and HIV-related chronic diseases, mainly atherosclerotic cardiovascular disease (CVD) (3–6). Some of the mechanisms responsible for this increased cardiovascular risk, in HIV-infected patients, involve HIV infection and inflammation, dyslipidemia, insulin resistance, as well as metabolic, and body composition changes induced by antiretroviral therapy (7–11). Moreover, non-HIV related risk factors, such as aging, can also contribute to the development of these metabolic alterations and risk factors (12–15).

Here we review how immune activation and insulin resistance contribute to the metabolic alterations observed in HIV-infected patients, and how these alterations increase the risk of developing CVD. In section Sources of Immune Activation and Insulin Resistance in HIV Patients, we discuss how HIV infection and inflammation, combination antiretroviral therapy (cART), and gut microbiota contribute to immune activation and insulin resistance. In section Consequences of Immune Activation and Insulin Resistance in HIV-Infected Patients, we review the clinical consequences of immune activation and insulin resistance, as well as how these processes are involved in the development of age-related metabolic diseases in HIV patients.

## Sources of immune activation and insulin resistance in HIV patients

The immune activation of HIV-infected patients, whether on cART or not, is usually accompanied by insulin resistance (16). In this section, the role of HIV infection and inflammation, cART, and gut microbiota in immune activation and molecular mechanism of insulin resistance are discussed.

### Effect of HIV infection and inflammation on insulin resistance

**Figure 1:**
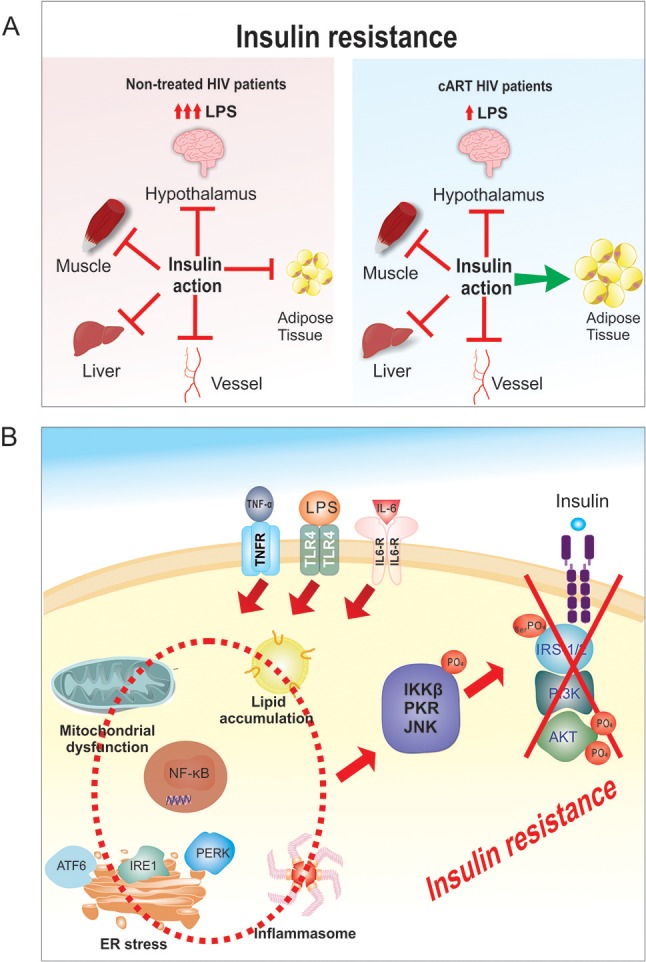
**(A)** Tissue specific insulin resistance. In HIV untreated patients there is severe insulin resistance with increased LPS and cytokines that involves liver, hypothalamus, muscle, vessels and adipose tissue. After cART treatment the insulin resistance is mild/moderate, with reduced LPS and cytokines that probably spares adipose tissue. **(B)** Molecular mechanism of LPS-induced insulin resistance. The increase in LPS circulating levels in HIV patients will induce an increase in circulating inflammatory cytokines, and these increases will activate TLR4, IL-6, and TNFalpha receptors, which will induce ER stress, mitochondrial dysfunction, activation of inflammasome and an increase in intracellular lipid accumulation. Then, there will be activation of PKR, JNK, and IKKβ/NF-κB pathways in liver, muscle, adipose tissue, macrophages and also in other tissues. The activation of these serine kinases (PKR, JNK, and IKKβ) will induce serine phosphorylation of the IRS1/2 and consequently a down regulation in insulin signaling.

It is generally accepted that there is a correlation between innate immune system activation and insulin resistance, which contributes to glucose metabolism dysregulation and dyslipidemia (8). Immune activation results in chronic inflammation, that varies in severity, and has been observed in untreated HIV patients and patients undergoing cART (17). However, untreated HIV-patients display an enhanced inflammatory state, which is characterized by high levels of proinflammatory cytokines, like tumor necrosis factor alpha (TNF-α), and interleukins (IL-6 and IL-1β), and is associated with a procoagulant state (12). Under these conditions, the insulin resistance is probably severe and could occur in the liver, muscle, and adipose tissue. In fact, severe insulin resistance in the adipose tissue (as observed in HIV untreated patients), may prevents adipose mass gain as described in mice (18–20) (Figure 1A).

In patients undergoing antiretroviral drug therapy, there is a decrease in proinflammatory cytokines, which do not completely return to normal, thus indicating that some level of inflammation persists (21). A variety of factors, such as: virus production, cytomegalovirus infection, regulatory T-cell loss, and/or lymphoid structure damage could contribute to this persistent inflammation (22, 23). There is still insulin resistance, but it is mild or moderate. As previously demonstrated in animal models of obesity (24, 25), less severe insulin resistance in adipose tissue allows normal or increased glucose uptake and lipid conversion in this tissue, favoring weight gain and contributing to explain the increase in visceral adipose tissue (VAT) in these patients (26, 27) (Figure 1A).

In HIV-treated patients, the activation of the innate immune system and insulin resistance is similar to what has been described in obesity and type 2 diabetes mellitus (DM2) (28, 29). The innate immune system and insulin signaling are integrated and toll-like receptors (TLRs), inducible nitric oxide synthase (iNOS), protein kinase R (PKR), c-Jun N-terminal kinases (JNK), and NF-κB are connected to the insulin receptor (IR) and its downstream signaling pathway IRS/PI3K/Akt. Upon activation of the innate immune system, proteins involved in insulin signaling pathways become posttranscriptionally modified, resulting in reduced insulin action (Figure 1B) (30, 31).

It is important to mention that bacterial lipopolysaccharide (LPS) from the Gram negative intestinal bacteria is continuously produced in the gut (secondary to death of Gram negative bacteria) and translocated to the circulation (32). This translocation depends on many factors including immune system, integrity of epithelia barrier, diet, and many other environmental factors. The increase in circulating LPS, through its own receptor -TLR4- induce the release of inflammatory cytokines that can contribute to insulin resistance (33, 34).

Previous data showed that there is an increase in LPS circulating levels in HIV patients, whether on treatment or not (35), which can induce at the same time TLR4 activation and endoplasmic reticulum (ER) stress (19, 36, 37). Then, there will be activation of PKR, JNK, and IKKβ/NF-κB pathways in liver, muscle, adipose tissue, macrophages, and also in other tissues. The activation of these serine kinases (PKR, JNK, and IKKβ) will induce serine phosphorylation of the insulin receptor substrate 1 and 2, and consequently a down regulation in insulin signaling (38, 39). The activation of NF-κB pathways in liver, adipose tissue, and macrophages will induce the production of proinflammatory cytokines (i.e., TNF-α, IL-1β, and IL-6), creating an inflammatory vicious cycle, which is even worst with the increased adiposity (40–43). Certainly, this aggravates inflammation and insulin resistance.

In addition to increase in circulating LPS and in proinflammatory cytokine TNF-α, IL-1β, and IL-6, the JNK and NF-κB pathways can also be activated by intracellular mechanisms that involve oxidative and endoplasmic reticulum (ER) stresses, activation of inflammasome and an increase in intracellular lipid accumulation (39, 44, 45) (Figure 1B). Additionally, augmented iNOS activity and the nitrosylation of insulin pathway proteins have been shown to promote insulin resistance (46, 47). In summary, an increase in LPS and proinflammatory cytokines and in intracellular mechanisms will activate serine kinases, resulting in insulin receptor substrate-1 (IRS-1) serine phosphorylation and insulin signal transduction inhibition (30, 31, 48).

### Effect of viral suppression on insulin resistance

Protease inhibitors (PI) or nucleoside analog reverse transcriptase inhibitors (NRTI) have been shown to induce insulin resistance, dyslipidemia, and lipodystrophy, and consequently increase cardiovascular risk (17, 49–51). These drugs increase the nuclear localization of SREBP-1 (sterol regulatory element-binding protein 1), which is a transcription factor that regulates the expression of genes associated with lipid synthesis (52). In the liver, these antiviral drugs can increase the levels of free intracellular cholesterol and lipids (53), which can affect aging and the immune system response. In the muscle and adipose tissue, these drugs can induce ER stress and reduce glucose transporter 4 (GLUT4) translocation to the plasma membrane (54, 55). The NRTIs also inhibit mitochondrial DNA-polymerase, respiratory chain function, and ATP production, ultimately leading to adipocyte death (56–60).

HIV patients undergoing cART exhibit a partial reversal of immune activation and inflammation. Additionally, cART reduces opportunistic infections and cardiovascular risk factors, which is likely a result of some reduction in inflammation (61), although residual markers of inflammation and coagulation remains elevated in ART-treated HIV-infected patients (62). In treated patients the dyslipidemia correlates better with C-reactive protein and IL-6 levels, rather than with CD4 count or HIV viral load, suggesting that immune activation has a central role in the development of dyslipidemia (63). While cART improves some of the observed alterations, it does not reverse the immune activation or chronic inflammation completely. In fact, patients undergoing cART still present a proinflammatory and prothrombotic state, accompanied by changes in the number and size of low-density lipoprotein (LDL) and high-density lipoprotein (HDL) particles, which increases the risk of these patients developing cardiovascular complications (62, 64–67).

### Effect of microbiota modulation on insulin resistance

While the progression from HIV infection to AIDS is primarily modulated by T cell activation and systemic inflammation, there is evidence that the gastrointestinal mucosa immune system also participates in this process (68). The human gut microbiota is mainly composed of four phyla: Firmicutes, Bacteroidetes, Actinobacteria, and Proteobacteria. The general population predominantly harbors Bacteroidetes, followed by Firmicutes; however, the composition of the gut microbiota is influenced by diet, age, geography, drugs, and cultural behaviors (69–72). Over the past 10 years, it is becoming clear that microbiota populations are modulated and may have a causal effect in more prevalent chronic conditions such as: obesity, diabetes, hypertension, and CVD (73).

Interestingly, HIV infection can also modulate the levels of bacteria of the gut microbiota. In fact, there is a decrease in the levels of the phylum *Bacteroidetes*, but some genders of this phylum, as *Prevotella*, increases when analyzed in treated and untreated patients (74–76). Such a change in the gut microbiota could result in increased tryptophan catabolism, chronic inflammation, and increased cardiovascular risk (77, 78). Additionally, an increase in *Prevotella* could augment circulating trimethylamine (TMA) levels, which is transformed into trimethylamine oxide (TMAO), and can have a role in the development of atherosclerosis (79).

Previous data showed that increased levels of choline and TMAO are associated with cardiovascular diseases (80). It is well known that ingested choline is transformed by gut microbiota in TMA, which enters portal circulation and in liver is converted in TMAO (80). It is interesting that fasting TMAO levels are independent predictor of atherosclerotic disease and high-risk mortality in coronary artery disease patients (81, 82). The mechanisms by which TMAO induces or accelerates atherosclerosis is not completely understood, it may involve macrophage activation and increase in foam cells and also modulation of platelet aggregation and adhesion (83). Moreover, besides promoting atherosclerosis lesion development, TMAO also aggravate pressure-overload heart failure in mice (84).

Recently, our group demonstrated that, in HIV patients, a close correlation exists between increased circulating LPS levels, a marker for intestinal permeability, and insulin resistance (35). The increased translocation of LPS and elevated serum levels induce the activation of the innate and adaptive immune systems (35, 85, 86). As discussed previously, in macrophages and most tissues, LPS binds to and activates TLR4, which initiates a complex cascade of signaling events, resulting in the downstream activation of the JNK and NF-kB pathways, and consequently insulin resistance and systemic inflammation. Additionally, LPS has also been shown to increase adipose tissue, which augments body weight gain (35, 87, 88). Moreover, in HIV-infected patients, elevated levels of LPS have been linked to endothelial dysfunction and adverse metabolic outcomes (35, 88–91).

It is important to mention that the effect of cART introduction in the modulation of gut microbiota is not completely understood. However, very recently Ji et al. showed that this modulation after cART was differentially correlated with the immune status, especially in patients with CD4 + T cell counts>300/mm^3^ (92). In these patients it was shown that the alpha diversity was correlated with CD4 + T cell counts, but the specific role of cART in increasing microbial diversity is still controversial (78, 92, 93). This correlation may explain the conflicting results in previous studies investigating alpha diversity in intestinal microbiota in HIV patients (93–96), indicating that this diversity is consequence of the immune status of the subjects. The immunological profile and cART seem to contribute together to alter the gut microbiota.

## Consequences of immune activation and insulin resistance in HIV-infected patients

**Figure 2:**
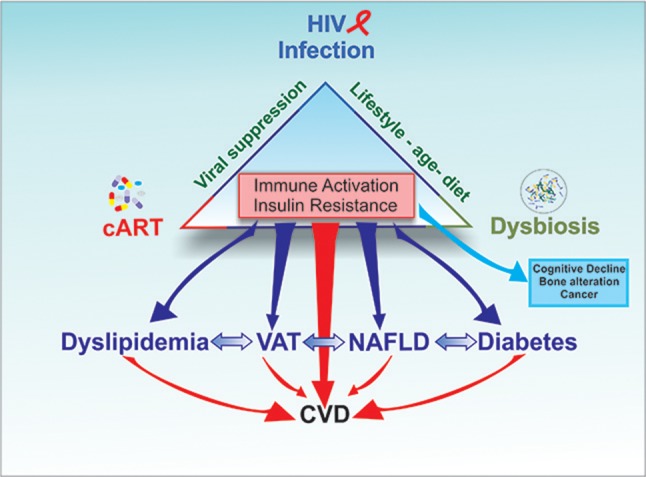
The triad HIV infection/inflammation, antiretroviral therapy (cART), and gut microbiota contribute to induce immune activation and insulin resistance. The clinical consequences of chronic immune activation and insulin resistance can contribute to increase Visceral Adipose Tissue (VAT), dyslipidemia, CVDs and non-alcoholic fat liver disease (NAFLD) as well as neurocognitive disorders, metabolic disorders, bone abnormalities and non-HIV associated cancers While the evolution of these complications depends on genetic and environmental factors, each condition has the potential of aggravating another, increasing the risk of CVD in HIV patients.

Chronic immune activation and insulin resistance can contribute to obesity, dyslipidemia, CVDs, and non-alcoholic fat liver disease (NAFLD) as well as neurocognitive disorders, metabolic disorders, bone abnormalities, and non-HIV associated cancers (12, 97, 98, 99). While the evolution of these complications depends on genetic and environmental factors, each condition has the potential of aggravating another (Figure 2).

### Obesity and lipodystrophy

Before the new generation of antiretroviral therapies, HIV was often associated with lipodystrophy, which is a marker for metabolic alterations and includes a broad spectrum of clinical alterations (100, 101). Previous studies showed that HIV infection severity was associated with an increased prevalence of lipodystrophy, which is secondary to HIV-infected macrophages infiltration and enhanced local inflammation in the adipose tissue (102, 103). In the past, the development of lipodystrophy was partially related to drugs (i.e., stavudine and zidovudine) included in the treatment regimen, but it is also influenced by age, CD4 levels, viral load, therapy duration, and race (especially caucasians). Remarkably, the new classes of cART and inhibitors (fusion, integrase, and entry) do not alter the metabolic parameters of fat distribution (1, 50).

Obesity and visceral adiposity are commonly observed in HIV-treated patients and are the result of factors associated with both traditional treatments and cART. As with most obese people, the increase in adipose tissue is associated with inflammatory and metabolic responses. Since many HIV patients have low muscle mass, excess adipose tissue may be present, even when the BMI is within the normal range. In fact, a recent study showed that when considering BMI, 60–70% of HIV-infected patients are considered overweight or obese (104–108). In most patients, there is an increase in visceral adipose tissue (VAT), which is usually indicative of a more deleterious metabolic profile (109–111).

In obese populations, metabolically healthy obesity is characterized by less VAT and reduced inflammation (112–115). These same more benign metabolic conditions have also been documented in some HIV infected patients considered overweight or obese. The reasons why some obese individuals, with or without HIV, have a more aggressive metabolic profile and associated risk factors are not completely understood, but it might involve adipocyte size and/or number, recruited inflammatory cells, hypoxia, and/or adiponectin levels (116–118).

The extrapolation of data from the obese population without HIV to those with HIV must be interpreted with care, since a large portion of the data related to adipose tissue dysfunction includes HIV patients with lipodystrophy. Although this alteration in fat distribution still occurs in treated HIV-infected patients, the prevalence of obesity is increasing, and in some patients, an association between obesity and lipodystrophy has been observed (119).

### Dyslipidemia

During the 80's and early 90's, before the introduction of antiretroviral therapy, dyslipidemia was evident in more severe HIV cases, and was characterized by high triglyceride (TG) levels and low levels of HDL-cholesterol (HDL-C) and LDL-cholesterol (LDL-C). Although the exact mechanisms that account for the development of this kind of dyslipidemia are not fully understood, there is data suggesting that it may be induced by insulin resistance resulting from HIV infection and inflammation (120–122). This observed pattern of dyslipidemia is not only observed in HIV patients, but is also detected in other infections and inflammations, and can become atherogenic if it persists (123, 124). In this regard, an increase in TNF-α level can impair the clearance of TGs and reduce the antilipolytic effect of insulin, thus stimulating lipolysis in HIV-patients with lipodystrophy.

Besides activation of the innate immune system and insulin resistance, which contributes to glucose metabolism dysregulation and dyslipidemia, other mechanisms may also contribute to explain this pattern of dyslipidemia. One such mechanism involves the adenosine-triphosphate binding cassette transporter A1 (ABCA1), a transmembrane transporter present in macrophages, which interacts with the HIV-produced accessory protein Nef. Under physiological conditions, ABCA1 shuttles cholesterol from macrophages (in peripheral tissues) to HDL, which was previously shown to be to reduce cardiovascular risk (87, 125). However, in HIV-infected patients, Nef downregulates ABCA1 expression and reduces the efflux of cholesterol to HDL. As a result, lipid accumulates inside the macrophage, and is transformed into foam cells, which is associated with atherosclerosis (125).

Another mechanism, that can contribute to the understanding of why this pattern of dyslipidemia is commonly observed in HIV-patients, is through the inhibition of an intracellular peroxisome protein, proliferator-activated receptor gamma (PPAR-γ) (126). This protein is critical for adipocyte differentiation, and is inhibited by the HIV viral protein, vpr. The inhibition of PPAR-γ blocks adipocyte differentiation, and leads to fatty acid accumulation and lipotoxicity. Moreover, HIV replication is also associated with an increase in fatty acid synthase (FAS) activity, which impacts fatty acid synthesis (127). These data suggest that in HIV-infected patients, not undergoing cART, there is an increase in fatty acid production that can contribute to the appearance of dyslipidemia and insulin resistance.

On the other side, it has been shown that cART not only suppresses HIV infection and reduces inflammation, but it also changes the dyslipidemia pattern, which is characterized by an increase in TGs and LDL-C, a reduction in HDL-C and maintenance of insulin resistance (128, 129). In fact, a recent meta-analysis study showed that cART patients have a higher risk of developing hypercholesterolemia and display higher TG levels than non-treated HIV patients (27). The previously described modulation of ABCA1 by Nef, which reduces the efflux of HDL, is also reversed by cART (130–132).

### Cardiovascular diseases and NAFLD

As previously mentioned, there is still an increased risk of CVD in the HIV-infected population, despite cART and the control of risk factors (1, 51). Even with the development of new antiretroviral drugs, the chronic immune activation and insulin resistance remain and contribute to this greater risk (12, 51). Also, recent data has shown that HIV-infected patients can present left ventricular systolic and diastolic dysfunction and myocardial fibrosis (133, 134).

Abnormal liver enzymes are also common in HIV-infected patients, despite the absence of alcohol consumption or viral hepatitis. These abnormalities have been associated with an increased prevalence of NAFLD and non-alcoholic steatohepatitis (NASH) (135). The actual prevalence of NAFLD and NASH in HIV-infected patients is not known because the methods used to define these alterations vary among studies (136). Recent data has shown that in HIV-infected patients, treated with cART, the prevalence of NAFLD is around 40%, and that these patients have a higher risk of developing NASH or cirrhosis than obese patients without HIV (137–141). These hepatic alterations are secondary to multiple factors, and immune activation, insulin resistance, cART, and aging are certainly involved in the process. It is also important to mention that NAFLD is a risk factor for diabetes and CVDs (142).

### Neurocognitive disorders, metabolic disorders, bone abnormalities, and non-HIV associated cancers

The HIV-infected population can develop behavioral abnormalities, motor dysfunction, and dementia (12). The clinical presentation can vary from mild neurocognitive disorders to severe HIV-associated dementia (143, 144). The prevalence of these abnormalities is ~50% (145), however, since the introduction of modern cART, the prevalence of severe forms of neurocognitive disorders has been dramatically reduced (146).

With regards to glucose metabolism, it is clear that current cART is much less metabolically toxic than previous therapies. However, HIV and cART are independently associated with glucose intolerance and diabetes (49). Again, while these abnormalities are secondary to multiple factors, glucose intolerance and diabetes are known to exacerbate the risk of these patients developing CVD.

Recent data has shown that HIV-infected patients have fracture rates three times higher than the control population (147, 148). In fact, decreased bone mineral density, osteopenia, and osteoporosis have been observed in these patients, and are probably related to immune activation and systemic inflammation, cART, low vitamin D, and/or aging.

The risk of some non HIV-associated cancers is 50% higher in HIV-infected patients than in non-infected patients (149, 150). For example, HIV infection is associated with a higher incidence of virus-related cancers such as: Kaposi sarcoma, lymphomas, and anal and liver cancer, which is most likely secondary to the poor immunological control of oncogenic viruses (12).

Overall, recent data showed that the mortality of HIV-infected people decreased in those below 65 years old, but increased after this age (112, 115), which is likely due to the increased risk of developing CVDs and dying from acute coronary syndrome. Furthermore, there is also an increase in the occurrence of coronary artery disease in young HIV-infected patients, when compared with the uninfected control population (113, 114, 151).

## Conclusion

The introduction of antiretroviral drugs has changed the clinical prognosis of HIV-infected patients and the deaths of these individuals are now related to atherosclerotic CVDs, rather than from the viral infection itself. However, HIV infection, cART, and intestinal microbiota are associated with immune activation and insulin resistance, which can lead to the development of a variety of diseases and disorders, especially with regards to CVDs. While lifestyle modifications and pharmaceutical interventions can be employed to treat these altered metabolic functions, the mechanisms involved in the development of these chronic complications remain largely unresolved. The elucidation and understanding of these mechanisms will give rise to new classes of drugs that will further improve the quality of life of HIV-infected patients, over the age of 50.

## Author contributions

MP and GR contributed to discussions and wrote, edited, and reviewed the article. DG, DM, HA, AO, and RP edited and reviewed the article. AS prepared to figures of the article, edited, and reviewed the article. MS contributed to discussions and wrote and reviewed the article. All authors approved the final version.

### Conflict of interest statement

The authors declare that the research was conducted in the absence of any commercial or financial relationships that could be construed as a potential conflict of interest.
